# Factors associated with burnout among the Belgrade University medical students

**DOI:** 10.2478/aiht-2025-76-3986

**Published:** 2025-12-30

**Authors:** Jovana Todorović, Kontstantinos Stratakis, Dejan Nešić, Ratko Tomašević, Zorica Terzić-Šupić

**Affiliations:** University of Belgrade Faculty of Medicine, Department of Social Medicine, Belgrade, Serbia; University of Belgrade Faculty of Medicine, Belgrade, Serbia; University of Belgrade Faculty of Medicine, Department of Medical Physiology, Belgrade, Serbia; Zemun Clinical Hospital Centre, Zemun, Serbia

**Keywords:** anxiety, Copenhagen Burnout Inventory, grade point average, Zung Self-Rating Anxiety Scale, anksioznost, Kopenhagenski upitnik o izgaranju, prosječna ocjena, Zungova samoocjenska ljestvica za depresiju

## Abstract

Although the 11^th^ Revision of the International Classification of Diseases defines burnout as an occupational syndrome, research has investigated it extensively in medical students. The aim of this cross-sectional study was to determine the prevalence of burnout among fifth-year medical students in Serbia along with the social, lifestyle, and health status characteristics associated with it. The study included 431 Belgrade University students attending classes in social medicine and took place in the last week of November 2024. According to the Copenhagen Burnout Inventory (CBI), the mean score was 43.67±15.81 and overall burnout prevalence 35.3 %. The prevalence of personal burnout was 35 %, of study-related burnout 36.9 %, and of faculty-related burnout 29 %. The multivariate logistic regression analysis of overall burnout showed positive association with the grade point average (OR: 4.02; 95 % CI: 2.12–7.64) and Zung Self-Rating Anxiety Scale score (OR: 1.14; 95 % CI: 1.06–1.22) and a negative association with the study engagement score (OR: 0.86; 95 % CI: 0.81–0.92). Our findings identify variables that need addressing to lower burnout prevalence among students. One is anxiety, which was significantly associated with burnout, and the other is study engagement, which was inversely associated with burnout. We believe that medical schools should provide mental health support programmes to address these and other potential issues.

## Abstract (hr)

Iako 11. revizija Međunarodne klasifikacije bolesti definira izgaranje kao sindrom koji je vezan za posao, velik broj istraživanja proveden je među studentima medicine. Cilj ovoga presječnog istraživanja bio je procjena prevalencije sindroma izgaranja među studentima pete godine medicine u Srbiji, zajedno sa socijalnim karakteristikama, karakteristikama životnog stila i zdravstvenog stanja povezanima s izgaranjem. U istraživanje, koje se provodilo zadnji tjedan mjeseca studenoga 2024., bio je uključen 431 student Medicinskoga fakulteta Sveučilišta u Beogradu koji su pohađali nastavu iz socijalne medicine. Prosječan rezultat na Kopenhagenskom upitniku o izgaranju (Copenhagen Burnout Inventory – CBI) bio je 43,67±15,81, a prevalencija ukupnog izgaranja bila je 36,3 %. Prevalencija osobnog izgaranja bila je 35 %, izgaranja povezanoga sa studiranjem 36,9 %, a onoga koji je povezan s nastavnicima Fakulteta bila je 29 %. Multivarijantna logistička regresijska analiza s ukupnim izgaranjem kao ishodišnom varijablom pokazala je pozitivnu povezanost izgaranja s prosječnom ocjenom tijekom studija (OR: 4,02; 95 % CI: 2,12 – 7,64) i rezultatom na Zungovoj samoocjenskoj ljestvici za depresiju (OR: 1,14, 95 % CI: 1,06 – 1,22) te negativnu povezanost s rezultatom na ljestvici angažmana tijekom studiranja (OR: 0,86; 95 % CI: 0,81 – 0,92). Rezultati ovog istraživanja upućuju na nužnost ciljanih preventivnih programa kako bi se smanjila prevalencija sindroma izgaranja među studentima. Prva je anksioznost, koja je značajno povezana s izgaranjem, a zatim i angažman tijekom studija, koji je negativno povezan s izgaranjem. Zaključno, programi potpore mentalnom zdravlju trebali bi biti dostupni na medicinskim fakultetima.

According to the 11^th^ Revision of the International Classification of Diseases (ICD-11), burnout is “a syndrome conceptualized as resulting from chronic workplace stress that has not been successfully managed. It is characterized by three dimensions: feelings of energy depletion and exhaustion, increased mental distance from one’s job or feelings of negativism or cynicism related to one’s job, and reduced professional efficacy” (1). Although this limits the phenomenon to a combination of feelings associated with one’s job and the workplace, there are a number of studies reporting burnout among university students, medical and healthcare in particular ( 2,3,4,5). The main contributors to the burnout among students include academic and family pressure, and the reported prevalence in this population varies from 7 % to 70 %, which makes it a significant public health issue (5). Such great differences in reported prevalence between the studies reflect differences in the instruments used, in burnout criteria, cut-off scores, and, importantly, differences in measured dimensions of burnout (5).

All university students are considered sensitive to the development of burnout, but the risk seems to be much more prominent in medical students, as the reported prevalence ranges from 17.6 % to 82 % ( 2, 3, 6, 7). A risk this high may be associated with the study year, pressure to complete all study obligations, emotional distress arising from patient care, and the feelings of lack of control (8).

Previous studies have considered a number of factors potentially contributing to burnout, including gender, low socioeconomic status, lifestyle (such as alcohol consumption, smoking, and physical activity), mental health issues (such as anxiety and depression), family relations, marital status, and study engagement ( 9,10,11,12,13,14,15,16,17,18,19,20,21 ).

Although there were two studies of burnout prevalence among Serbian medical students, one dealt only with gender differences (10), showing significantly higher burnout in male than female students (19 % vs 12.8 %, respectively), in contrast to the US nationwide findings (9), and another (22) reporting the overall 32.2 % prevalence as part of the validation of the Serbian version of the Study Burnout Inventory (SBI) and the Copenhagen Burnout Inventory (CBI) for students, but which did not examine possible associations with contributing factors.

The aim of our study was therefore to address this gap by not only determining burnout prevalence among medical students in Serbia but also by establishing associations with a number of factors referred to above.

## PARTICIPANTS AND METHODS

This cross-sectional study was conducted at the University of Belgrade Faculty of Medicine during the last week of November 2024 and included 431 fifth-year students (response rate 100 %) attending the classes in Social Medicine, who were all informed about the study, including its aim and instrument, and who gave consent for participation. The study was approved by the Ethics Committee of the University of Belgrade, Faculty of Medicine (approval No. 25/IX-4 of 16 September 2024).

The study instrument was a questionnaire that consisted of the following sections: 1) participant demographics socioeconomic characteristics, and academic achievement; 2) lifestyle and health status; 3) study engagement scale; 4) Multidimensional Scale of Perceived Social Support (MSPSS); 5) Patient Health Questionnaire-9 (PHQ-9); 6) Zung Self-Rating Anxiety Scale (SAS); 7) International Physical Activity Questionnaire, short form (IPAQ-SF); and 8) Copenhagen Burnout Inventory (CBI).

Additionally, we measured participants’ grip strength on dominant and non-dominant hand in newtons (N) and then classified it for both genders into quartiles.

Participant demographics and socioeconomic characteristics included gender (male/female), age (years), current residence (own apartment/rented apartment/student dorm/parental home/other), provenance (urban/rural), financial status (poor/average/good), family relations (poor/average/good), partner status (single/with partner). Academic achievement was evaluated as grade point average (GPA).

Lifestyle and health status included self-rated health (poor/average/good), height and weight (from which we calculated the body mass index in kg/m^2^), smoking status (tobacco/e-cigarettes/heated tobacco products), alcohol use (including binge drinking), cannabis use, time spent on social media per day, time spent playing video games per day, time spent using mobile phone per day, and use of anxiety medicines in the past 12 months.

The study engagement scale, described in detail elsewhere (12), consisted of nine items assessing vigour, dedication, and absorption in study. All items are rated on a six-point Likert scale from 1 (completely disagree) to 6 (completely agree) and the score ranges from 9 to 54, with higher scores indicating higher engagement. Cronbach’s alpha for this scale was 0.779.

MSPSS was used to determine subjective assessment of social support as described in detail by Zimmer and McDonough (23). It consists of 12 items covering social support from the partner (if any), friends, and family. Each item is scored on a seven-point Likert scale from 1 (completely disagree) to 7 (completely agree), and the averaged total ranges from 1 to 7, with higher scores indicating higher perceived social support. Cronbach’s alpha for this scale was 0.934.

Depression was assessed with a nine-item PHQ-9 on a four-point Likert scale from 0 (never) to 3 (nearly every day) as described elsewhere (24). The scores can range from 0 to 27, with higher scores indicating more severe depressive symptoms. Cronbach’s alpha for this scale was 0.867.

Anxiety experienced over the past seven days was determined with the 20-item Zung SAS on a four-point Likert scale from 1 (never or rarely) to 4 (most of the time) as described elsewhere (25). The total can range from 20 to 80, and higher scores indicate higher levels of anxiety. Cronbach’s alpha for this scale was 0.720.

Physical activity was determined with the six-item IPAQ-SF (26) calculating energy consumption through the self-reported time spent in vigorous and moderate physical activity and walking per week, which is expressed in metabolic equivalent of a task minutes (MET minutes) per week.

Burnout symptoms were determined with the Serbian version of CBI adapted for students to rate three domains – personal burnout, study-related burnout, and faculty-related burnout (22) – on a five-point Likert scale. The scores were then converted to percentages, as follows: 0 (never) to 0 %; 1 (rarely) to 25 %, 2 (sometimes) to 50 %; 3 (often) to 75 %; and 4 (always) to 100 %. The score for each domain was calculated as the average of all item scores in that domain. The total score is the average of the three domains and served to establish the cut-off point between participants with burnout (total score >50 %) and those without burnout (total score ≤50 %) as described elsewhere (22, 28). The original CBI (27) coincides with the Serbian version in personal burnout, but differs in the other two domains, as it measures work-related and client-related burnout. The Cronbach’s alpha for our CBI was 0.910, more specifically 0.890 for personal burnout, 0.802 for study-related burnout, and 0.907 for faculty-related burnout.

### Statistical analysis

Differences between categorical variables were determined with the chi-squared test and those between the numerical variables with the Mann-Whitney *U* test, as Kolmogorov-Smirnov test showed no normal data distribution.

All significant differences (p<0.05) entered multivariate logistic regression analysis against overall, personal, study-related, and faculty-related burnout as the outcome variables. All analyses were done using the IBM SPSS Statistics version 22.0 (Armonk, NY, USA).

## RESULTS

The prevalence of overall burnout (average of all three domains in CBI) among all students was 35.3 % (152/431), while the prevalence of personal burnout was 35 % (151/431), of study-related burnout 36.9 % (159/431), and faculty-related burnout 29 % (125/431). We found no gender differences in either the overall or study- and faculty-related burnout.

Table 1 shows the differences in self-reported data between the students with and without burnout. Not all students answered all questions, so the percentages given here refer to within-group prevalences. Participants with burnout had a significantly lower prevalence of reporting family relationships and health as good, and significantly higher prevalence of reporting having used anxiety medicines in the past 12 months. In addition, they reported higher GPA, less time spent on mobile phone per day and had higher PHQ-9 and Zung SAS scores and lower study engagement, MSPS, and physical activity scores.

**Table 1:** Differences between participants with and without overall burnout^*^

**Characteristic**		**With burnout**	**Without burnout**	p-value**
Provenance N (%)	Urban	132 (88.6)	243 (91.4)	
	Rural	17 (11.4)	23 (8.6)	0.360
Gender N (%)	Male	42 (27.6)	83 (31.2)	
	Female	110 (72.4)	183 (68.8)	0.443
Age (in years) (mean±SD)		23.45±1.45	22.22±16.82	0.086
BMI (in kg/m^2^) (mean±SD)		23.07±3.82	22.77±3.39	0.873
Current residence N (%)	Own apartment	34 (22.4)	66 (24.7)	
	Rented apartment	28 (18.4)	63 (23.6)	
	Student dorm	21 (13.8)	48 (18.0)	
	Parental home	65 (42.8)	85 (31.8)	
	Other	4 (2.6)	5 (1.9)	0.205
GPA (mean±SD)		8.87±0.68	8.57±0.72	**0.015**
Smoking tobacco N (%)	Yes	40 (26.8)	54 (21.2)	
	No	109 (73.2)	201 (78.8)	0.193
Smoking e-cigarettes N (%)	Yes	18 (11.8)	36 (13.6)	
	No	134 (88.2)	229 (86.4)	0.610
Heated tobacco products N (%)	Yes	15 (9.9)	32 (12.1)	
	No	137 (90.1)	233 (87.9)	0.493
Alcohol use N (%)	Yes in the past year, but not in the past 30 days	36 (23.7)	57 (21.3)	
	Yes, in the past 30 days	107 (70.4)	185 (69.3)	
	No	9 (5.9)	25 (9.4)	0.434
Binge drinking N (%)	Yes in the past year, but not in the past 30 days	50 (33.1)	65 (24.4)	
	Yes, in the past 30 days	52 (34.4)	104 (39.1)	
	No	49 (32.5)	97 (36.5)	0.163
Cannabis use N (%)	Yes	25 (16.4)	30 (11.2)	
	No	127 (83.6)	237 (88.8)	0.129
Time spent on social media per day (mean±SD)		2.75±1.64	2.97±1.64	0.954
Time spent playing video-games per day (mean±SD)		0.51±1.49	0.32±0.78	0.874
Time spent using mobile phone per day (mean±SD)		34.59±41.65	36.66±43.69	**0.027**
Subjective financial status N (%)	Poor	4 (2.6)	4 (1.5)	
	Average	51 (33.6)	89 (33.3)	
	Good	97 (63.8)	174 (65.2)	0.712
Family relations N (%)	Poor	11 (7.2)	10 (3.7)	
	Average	32 (21.1)	31 (11.6)	
	Good	109 (71.7)	226 (84.6)	**0.006**
Health N (%)	Poor	7 (4.7)	4 (1.5)	
	Average	31 (20.7)	28 (10.5)	
	Good	112 (74.7)	235 (88.0)	**0.002**
Relationship status N (%)	Single	94 (61.8)	140 (52.4)	
	With partner	58 (38.2)	127 (47.6)	0.062
Anxiety medicines in the past 12 months N (%)	Yes	60 (39.5)	64 (24.0)	
	No	92 (60.5)	203 (76.0)	**0.001**
Strength on the dominant hand N (%)	<25^th^ percentile	35 (23.0)	71 (26.7)	
	25^th^ to 75^th^ percentile	73 (48.0)	137 (51.5)	
	>75^th^ percentile	44 (28.9)	58 (21.8)	0.251
Strength on the non-dominant hand N (%)	<25^th^ percentile	37 (24.3)	74 (27.8)	
	25^th^ to 75^th^ percentile	76 (50.0)	126 (47.4)	
	>75^th^ percentile	39 (25.7)	66 (24.8)	0.738
PHQ-9 score (mean±SD)		9.72±5.27	6.46±5.28	**0.001**
Zung SAS score (mean±SD)		39.98±8.41	32.85±7.13	**0.001**
Study engagement score (mean±SD)		30.63±8.02	36.43±5.92	**0.001**
MSPSS score (mean±SD)		5.60±1.07	6.37±0.79	**0.001**
Physical activity (MET minutes/week; mean±SD)		2629.48±1927.84	2851.56±1860.09	**0.001**

* calculated as average of the three CBI domain scores (overall burnout cut-off score >50 %);

** significant differences are in boldface; BMI – body mass index (kg/m^2^);

CBI – Copenhagen Burnout Inventory, Serbian version adapted for students; GPA – grade point average; MET – metabolic equivalent of a task; MSPSS – Multidimensional Scale of Perceived Social Support; PHQ-9 – Patient Health Questionnaire-9; SAS – Self-Rating Anxiety Scale; SD – standard deviation of the mean

As for the differences between students with and without personal burnout, they were significant for the female gender (78.8 % vs 65.8 %; p=0.005), alcohol consumption in the past year (28.5 % vs 19.2 %; p=0.005), binge drinking in the past year (33.3 % vs 24.0 %), binge drinking in the past month (28.7 % vs 42.2 %), rating family relationships as good (72.2 % vs 84.4 %; p=0.010), rating health as good (75.2 % vs 87.7 %; p<0.001), use of anxiety medicines in the past 12 months (41.7 % vs 22.5 %; p<0.001), measured strength on the dominant hand >75^th^ percentile adjusted for gender (30.5 % vs 20.7 %; p=0.036), Zung SAS scores (40.97±7.85 vs 32.20±6.93; p<0.001), PHQ-9 scores (10.10±4.93 vs 6.18±5.22; p<0.001), study engagement scores (31.94±8.27 vs 35.82±6.35; p<0.001), and MSPSS scores (5.72±1.06 vs 6.32±0.85; p<0.001).

Differences between students with and without study-related burnout were significant in the prevalence of cannabis use (17.6 % vs 10.6 %; p=0.040), anxiety medicine use in the past 12 months (39.0 % vs 23.5 %; p<0.001), Zung SAS score (39.14±8.92 vs 33.05±7.15; p<0.001), PHQ-9 score (9.46±5.34 vs 6.48±5.21; p<0.001), study engagement score (30.77±8.19 vs 36.37±5.85; p<0.001), and MSPSS score (5.79±1.00 vs 6.28±0.91; p<0.001).

Differences between students with and without faculty-related burnout were significant in having a partner (64.8 % vs 52.3 %; p=0.018), time spent using mobile phone per day (40.75±59.50 vs 34.42±33.99; p=0.008), Zung SAS score (37.84±8.50 vs 33.99±8.07; p=0.003), PHQ-9 score (8.18±5.67 vs 7.19±5.34; p=0.019), study engagement score (31.11±8.10 vs 35.95±6.39; p<0.001), and MSPSS score (5.84±1.04 vs 6.24±0.91; p=0.021).

Table 2 shows the results of multiple regression analysis revealing associations between student variables and overall, personal, study-related, and faculty-related burnout. Overall burnout as an outcome variable has a significant positive association with GPA and Zung SAS score and negative association with the study engagement score.

**Table 2:** Associations between student variables and burnout* as outcome variable obtained with multivariate logistic regression analysis**

**Characteristic**		**Overall burnout OR (95 % CI)**	**Personal burnout OR (95 % CI)**	**Study-related burnout OR (95 % CI)**	**Faculty-related burnout OR (95 % CI)**
Gender	Male	/	1.0	/	/
	Female	/	**2.12 (1.13–3.96)**	/	/
GPA (mean±SD)		**4.02 (2.12–7.64)**	**/**	/	/
Alcohol use	Yes in the past year, but not in the past 30 days	/	**8.15 (2.16–30.78)**	/	/
	Yes, in the past 30 days	/	**5.20 (1.40–19.24)**	/	/
	No	/	1.0	/	/
Binge drinking	Yes in the past year, but not in the past 30 days	/	**0.48 (0.24–0.98)**	/	/
	Yes, in the past 30 days	/	**0.39 (0.19–0.82)**	/	/
	No	/	1.0	/	/
Cannabis use	Yes	/	/	1.54 (0.74–3.19)	/
	No	/	/	1.0	**/**
Time spent playing video games per day (mean±SD)		/	/	/	/
Time spent using mobile phone per day (mean±SD)		0.99 (0.98–1.01)	1.00 (0.99–1.01)	**/**	0.99 (0.99–1.01)
Family relations	Poor	1.79 (0.31–10.50)	1.97 (0.53–7.33)	/	/
	Average	1.05 (0.33–3.28)	1.06 (0.31–1.56)	/	/
	Good	1.0	1.0	**/**	**/**
Self-rated health status	Poor	1.41 (0.20–10.05)	0.23 (0.03–1.56)	/	/
	Average	2.37 (0.84–6.65)	**0.15 (0.02–0.91)**	/	/
	Good	1.0	1.0	**/**	**/**
Relationship status	Single	/	/	/	1.0
	With partner	/	/	/	1.58 (0.98–2.56)
Anxiety medicine use in the past 12 months	Yes	1.35 (0.57–3.18)	**2.25 (1.26–3.99)**	**2.03 (1.22–3.36)**	
	No	1.0	1.0	1.0	
Strength on the dominant hand	<25^th^ percentile		1.0		
	25^th^to 75^th^ percentile	/	1.41 (0.72–2.75)	/	/
	>75^th^ percentile	/	**2.28 (1.06–4.89)**	/	/
PHQ-9 score (mean±SD)		1.01 (0.92–1.10)	1.01 (0.95–1.07)	1.04 (0.99–1.09)	0.98 (0.93–1.04)
Zung SAS score (mean±SD)		**1.14 (1.06–1.22)**	**1.13 (1.07–1.20)**	**1.05 (1.01–1.10)**	1.04 (0.99–1.09)
Study engagement score (mean±SD)		**0.86 (0.81–0.92)**	**0.92 (0.87–0.96)**	**0.89 (0.86–0.93)**	**0.91 (0.88–0.94)**
MSPSS score (mean±SD)		0.68 (0.43–1.10)	**0.71 (0.50–0.99)**	0.83 (0.65–1.05)	0.96 (0.75–1.22)
Physical activity (MET minutes/week; mean±SD)		1.00 (1.00–1.00)	/	/	/

* calculated as average of the three CBI domain scores (overall burnout) or the average of each domain score (burnout cut-off score >50 %);

** significant associations are in boldface;

CBI – Copenhagen Burnout Inventory, Serbian version adapted for students; CI – confidence interval; GPA – grade point average; MET – metabolic equivalent of a task; MSPSS – Multidimensional Scale of Perceived Social Support; OR – odds ratio; PHQ-9 – Patient Health Questionnaire-9; SAS – Self-Rating Anxiety Scale; SD – standard deviation of the mean

Personal burnout, in turn, has a significant positive association with female gender, alcohol use in the past year, alcohol use in the past month, anxiety medicine use in the past 12 months, strength on the dominant hand above 75^th^ percentile adjusted for gender, and Zung SAS score. Multivariate log regression analysis also reveals significant negative association with binge drinking in the past year, binge drinking in the past month, self-rated health, study engagement score, and MSPSS score.

As for study-related burnout, regression analysis shows significant positive association with anxiety medicine use in the past 12 months and Zung SAS score and a significant negative association with study engagement score.

Faculty-related burnout, in turn, shows only the significant negative association with the study engagement score.

## DISCUSSION

Overall burnout prevalence in our study is almost identical to the 37.23 % reported by a meta-analysis of 42 studies in medical students across the world (6) and is higher than the prevalence reported among the fifth-year medical students from five universities in Serbia in 2019, which was 32.2 % (22). This difference may be owed to the COVID-19 pandemic that occurred in the meantime, and which, according to some studies in physicians and students (29, 30), may have aggravated burnout symptoms. The difference may also be owed to the fact that the earlier Serbian study covered five universities across Serbia, while this one is limited to students from Belgrade University alone.

Overall burnout in our study is positively associated with GPA, with students reporting higher GPA having more than four times higher likelihood for overall burnout. This finding stands in contrast to a study by Madigan and Curran (31), which found burnout associated with worse academic outcomes, most likely owing to inability and unwillingness to dedicate time and effort to studying. Overall burnout in our study is also positively associated with Zung SAS score, which is also true for personal and study-related burnout. The increase in risk per each score point on the Zung SAS ranges from 5 % for study-related burnout to 14 % for overall burnout. Both findings point to higher academic pressure to achieve the desired outcomes or to a culture of competitiveness, which is also the gist of an earlier study in Kragujevac University medical students (32).

In contrast, the likelihood of burnout is inversely associated with study engagement in our study, indicating that students with higher vigour, dedication, and absorption in studying have lower likelihood of burnout. This inverse association is visible in overall burnout and all three domains, with the likelihood decreasing from 8 % for personal burnout to 14 % for overall burnout. These findings are in line with the concept of study engagement being the opposite of burnout (12, 33).

Dominant hand grip strength in the 75^th^ percentile (adjusted for gender) is another parameter that seems to double the likelihood of personal burnout. Hwang et al. (34) reported an association between hand grip strength and higher levels of self-perceived stress among Korean female students. Considering that two-thirds of medical students in our study are female, this prevalence may explain our findings. Furthermore, female gender doubled the likelihood of personal burnout in our study. Female students may have worse mental health outcomes associated with body image, and therefore may perceive more stress, which may be measured as a personal burnout (35).

Alcohol consumption in the past year was associated with eight times higher likelihood of personal burnout in our study, while alcohol consumption in the past month was associated with more than five times higher likelihood, which is in line with earlier reports (36, 37) and suggests that university students may resort to alcohol to relieve stress.

In contrast, we have found the opposite association between binge drinking and personal burnout. Binge drinking behaviour may rather be associated with social occasions and peer pressure to engage in this type of behaviour and not as a coping mechanism for stress relief (38).

Another, quite expected, inverse association we found was between social support (MSPSS score) and personal burnout, in line with a meta-analysis in Chinese students (39).

The main limitation of our study is its cross-sectional design, as it does not allow us to establish the causal relationship between the variables. In addition, the data are self-reported, which may have resulted in under- or overestimation of burnout and its prevalence. We also limited our study to fifth-year medical students, which makes our findings less generally applicable. However, one of the main strengths is the high response rate and a relatively representative sample, at least for the fifth-year medical students.

## CONCLUSION

Burnout remains a significant public health issue among healthcare professionals, and our study shows its high prevalence among future physicians, which suggests that the problems may start as early as medical training. Our findings also identify variables that need addressing to lower the burnout prevalence this high. One is anxiety, which was significantly associated with overall, personal, and study-related burnout, and the other is study engagement, which was inversely associated with burnout. This emphasises the significant role of positive learning environment in relieving anxiety symptoms and promoting higher engagement with the curriculum. Medical schools should provide structured mental health support and even screening for anxiety and burnout symptoms, along with programmes that improve resilience of their students.

Future research should focus on the interaction between anxiety symptoms, study engagement levels, and burnout for us to better understand how psychological factors on one hand and academic factors on the other interact during medical education. It should also expand to longitudinal studies, examining changes throughout medical training to identify factors significantly contributing to burnout in more detail. Research should also focus on tailored preventive interventions, by assessing different approaches that improve study engagement and decrease study-related stress.
